# Supplementary material for: EnzML: multi-label prediction of enzyme classes using InterPro signatures
Source: BMC Bioinformatics. 2012 Apr 25;13:61. doi: 10.1186/1471-2105-13-61 (PMC3483700; doi:10.1186/1471-2105-13-61)
Supplement: Addtional file 5 — The Java code to format the data files, evaluate and predict. The file enzml_java_code.tar.gz contains the Java code used to format database data to ARFF and XML formats, to execute cross and train-test (jackknife) evaluations and to record evaluation results to database. More information is included in the readme.txt file and the Javadoc files. The code can be used with a MySQL database. To use a different database software, other JDBC drivers might be required. [file 1471-2105-13-61-S5.gz › java_code/enzml2011/doc/uk/ac/ed/inf/taweka/mulan/MulanInstancesFiller.html]

MulanInstancesFiller


---


|  |  |  |
| --- | --- | --- |
| **Overview** \| **Package** \| **Class** \| **Use** \| **Tree** \| **Deprecated** \| **Index** \| **Help** | |  |
| **PREV CLASS**   NEXT CLASS | **FRAMES**    **NO FRAMES**     **All Classes** |  |
| SUMMARY: NESTED \| FIELD \| CONSTR \| METHOD | DETAIL: FIELD \| CONSTR \| METHOD |  |


---


## uk.ac.ed.inf.taweka.mulan Class MulanInstancesFiller

```
java.lang.Object
  uk.ac.ed.inf.taweka.weka.InstancesFiller
      uk.ac.ed.inf.taweka.mulan.MulanInstancesFiller
```

---

``` public class MulanInstancesFiller extends InstancesFiller ```

Class

**Version:**
:   7 May 2010

**Author:**
:   Luna De Ferrari luna.deferrari-at-ed.ac.uk

---

**Constructor Summary**

`MulanInstancesFiller(MulanDataSetGenerator dataSetGenerator)`


| **Method Summary** | |
| --- | --- |
| `weka.core.SparseInstance` | `fillInstance(java.lang.String thisInstanceName, weka.core.SparseInstance instance)`             Consider all attributes! there is no last class attribute. |
| `MulanDataSetGenerator` | `getDataSetGenerator()`             get instances map public TreeMap getInstanceNameObjectMap() { return m\_instancesMap; } |

**Methods inherited from class uk.ac.ed.inf.taweka.weka.InstancesFiller**

`getInstancesNames`

**Methods inherited from class java.lang.Object**

`equals, getClass, hashCode, notify, notifyAll, toString, wait, wait, wait`

**Constructor Detail**

### MulanInstancesFiller

```
public MulanInstancesFiller(MulanDataSetGenerator dataSetGenerator)
```


**Method Detail**

### fillInstance

```
public weka.core.SparseInstance fillInstance(java.lang.String thisInstanceName,
                                             weka.core.SparseInstance instance)
```

:   Consider all attributes! there is no last class attribute.

    :   **Overrides:**: `fillInstance` in class `InstancesFiller`
    :   **Parameters:**: `thisInstanceName` - instance name: `instance` - a weka instance **Returns:**: a filled instance

---


### getDataSetGenerator

```
public MulanDataSetGenerator getDataSetGenerator()
```

:   **Description copied from class: `InstancesFiller`**
:   get instances map public TreeMap
    getInstanceNameObjectMap() { return m\_instancesMap; }

    :   **Overrides:**: `getDataSetGenerator` in class `InstancesFiller`


---


|  |  |  |
| --- | --- | --- |
| **Overview** \| **Package** \| **Class** \| **Use** \| **Tree** \| **Deprecated** \| **Index** \| **Help** | |  |
| **PREV CLASS**   NEXT CLASS | **FRAMES**    **NO FRAMES**     **All Classes** |  |
| SUMMARY: NESTED \| FIELD \| CONSTR \| METHOD | DETAIL: FIELD \| CONSTR \| METHOD |  |


---
